# A Case of Transient Global Amnesia Triggered by Sexual Intercourse

**DOI:** 10.7759/cureus.30564

**Published:** 2022-10-21

**Authors:** Esraa Askar, Harsimran Gill, Neeraj Singh

**Affiliations:** 1 Internal Medicine, Northwell/Hofstra Zucker School of Medicine, Long Island Jewish Forest Hills, USA; 2 Neurology, Northwell/Hofstra Zucker School of Medicine, Long Island Jewish Forest Hills, USA

**Keywords:** cognitive, cva, intercourse, mri, transient global amnesia

## Abstract

Transient global amnesia (TGA) is described as a sudden onset of anterograde and retrograde amnesia. In this case report, we present a case of a 52-year-old man who came to our hospital experiencing sudden-onset confusion and memory loss an hour after engaging in sexual activity with his wife. Before this, the patient had no previous medical history. Four hours following the event, a non-contrast computed tomography (CT) scan was performed and showed no signs of any intracranial pathology. In addition, T1-weighted and T2-weighted magnetic resonance imaging (MRI) was performed 13 hours following the incident and demonstrated punctate focus of restricted diffusion in the medial right temporal lobe in the region of the hippocampus. A diagnosis of transient global amnesia was made after other causes were ruled out. We present this rare case of transient global amnesia (TGA), highlighting sexual intercourse as a probable triggering component of the disease and demonstrating how crucial it is to rule out other severely morbid conditions before managing TGA.

## Introduction

Transient global amnesia (TGA) is defined as a sudden onset of anterograde and retrograde amnesia, which is often accompanied by impairment in executive function and recognition, can last for up to 24 hours, and is not linked to any other neurological impairment [1-3].

Mild neurological, psychological, and vegetative symptoms are possible following the incident, and they can continue for a few days. Migraine history, cardiovascular risk factors, such as ischemic heart disease, and psychophysical stress are the main risk factors for TGA [4,5].

The cause of transient global amnesia has yet to be discovered, despite its widespread acceptance as a clinical entity. Even though TGA is a condition with diagnostic criteria, it is considered a diagnosis of exclusion. It can occur among people who are typically healthy and do not have any comorbidities, risk factors, or triggering factors. Transient global amnesia has been linked to vascular risk factors, migraine, and epilepsy.

## Case presentation

A 52-year-old man presented with the complaint of memory loss and confusion, which started at 1:00 am when he was planning to go to bed. He had driven from North Carolina to New York the day before, but he had forgotten part of the journey. He last remembered being at a convenience store in Baltimore and next remembered being at his home in New York. He presented to the emergency department (ED) at 3:00 am accompanied by his wife who acted as his primary historian. The wife stated that he complained of a headache after they had sexual intercourse at 12:15 am, and then she noticed that he was confused. She did not observe the patient having any loss or alteration of consciousness. There was no head injury, weakness in any of the limbs, changes in vision, chest pain, shortness of breath, vomiting, or any other physical complaints. Additionally, there was no prior history of traumatic brain damage, stroke, dementia, seizures, psychiatric disease, or other incidents resembling this event. After the initial assessment in the emergency department, the patient was unable to identify the current date, president, or his address. There was no evidence of facial asymmetry or unilateral weakness, and the level of blood glucose was found to be 114 mg/dL. He did not have any previous medical conditions, and he was not taking any medications. He denied any history of smoking, alcohol, or recreational drug use.

The patient's vital signs during the ED physical examination showed blood pressure of 173/95 mmHg, heart rate of 93 beats per minute, body temperature of 99.1°F, respiratory rate of 18 breaths per minute, and oxygen saturation of 99% on room air. The patient was conscious, coherent, and alert, with a normal cranial nerve examination, gait, sensation, and motor strength throughout all four extremities. The Glasgow Coma Scale was 15, and the Cincinnati Prehospital Stroke Scale was negative.

**Figure 1:**
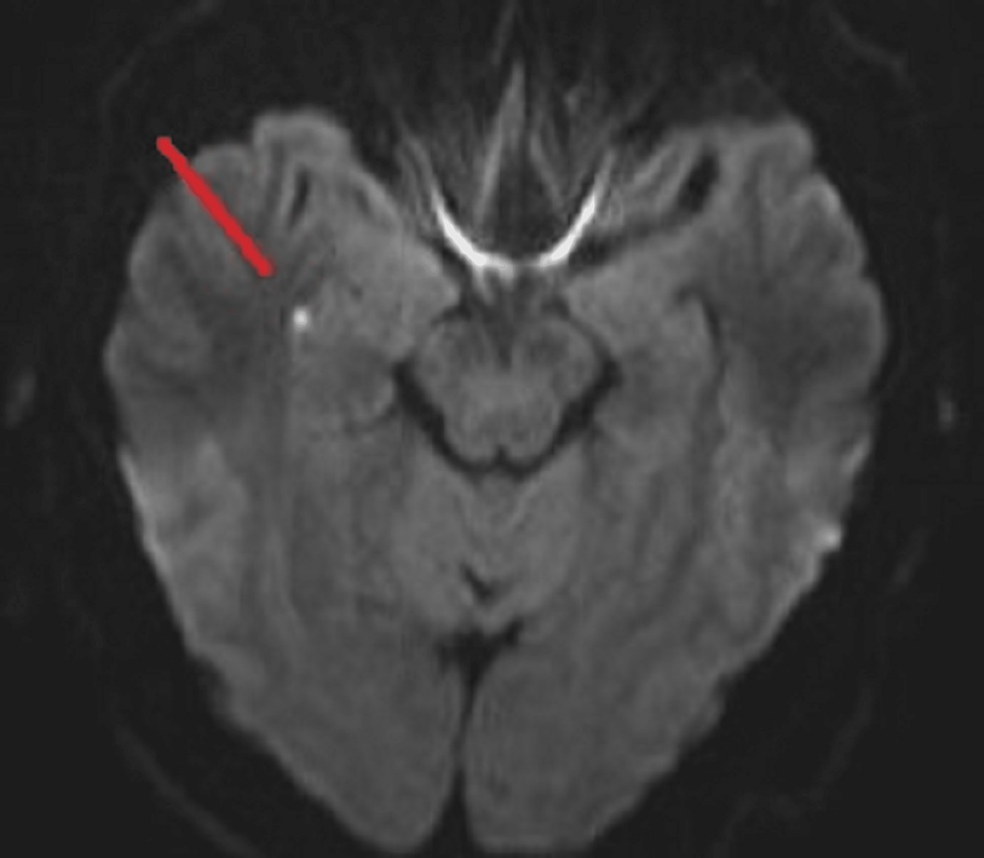
Magnetic resonance diffusion-weighted imaging (DWI) showing a punctate area of diffusion restriction in the medial right temporal lobe 13 hours after the episode

**Figure 2:**
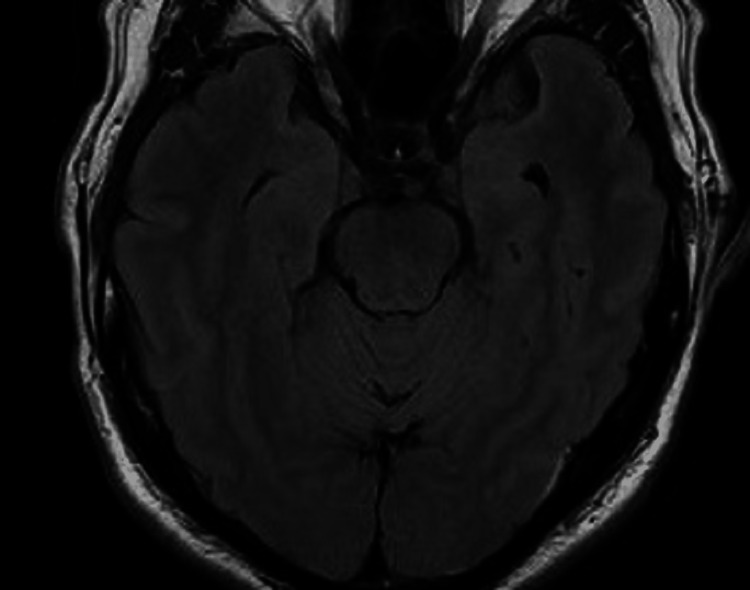
MRI fluid-attenuated inversion recovery (FLAIR) sequence 13 hours after the episode

**Figure 3:**
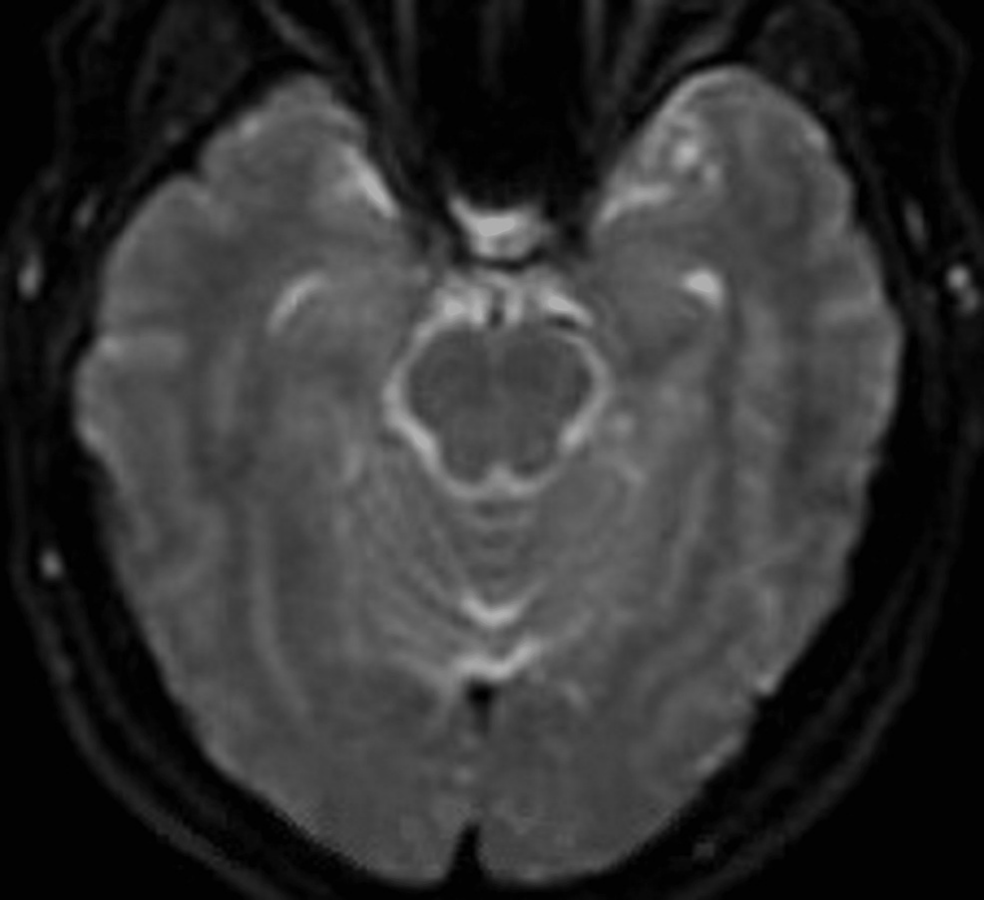
MRI T2-weighted sequence 13 hours after the episode

Complete blood count (CBC), complete metabolic panel (CMP), thyroid-stimulating hormone (TSH), lipid panel, polymerase chain reaction (PCR) test for coronavirus disease 2019 (COVID-19), and serum and urine toxicology screens were all unremarkable at the time of admission. The patient was treated empirically with aspirin 81 mg daily, clopidogrel 75 mg daily, and atorvastatin 80 mg daily for secondary stroke prevention. Four hours following the event, a non-contrast computed tomography (CT) scan was performed and showed no signs of any intracranial pathology. In addition, a magnetic resonance imaging (MRI) brain scan was performed 13 hours after the episode and demonstrated punctate focus of restricted diffusion in the medial right temporal lobe in the region of the hippocampus (red arrow in Figure 1, Figure 2, Figure 3).

Twenty-four hours after the incident, the patient was reevaluated. He was oriented to person and place but not to time. The patient had improved by this time with little to no intervention. Fifteen hours after the incident, an electroencephalogram (EEG) revealed no seizure activity and no focal slowing. At the time of discharge, 48 hours after the incident, the patient was found to be alert and oriented to person, place, and time, with no new neurological deficits.

**Figure 4:**
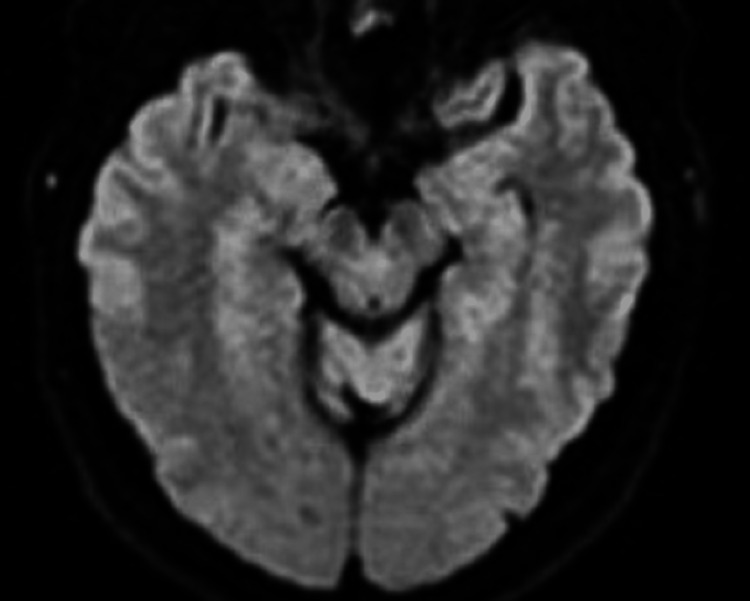
Magnetic resonance diffusion-weighted imaging (DWI) showing the same area, without any apparent residual findings, eight months after the episode

**Figure 5:**
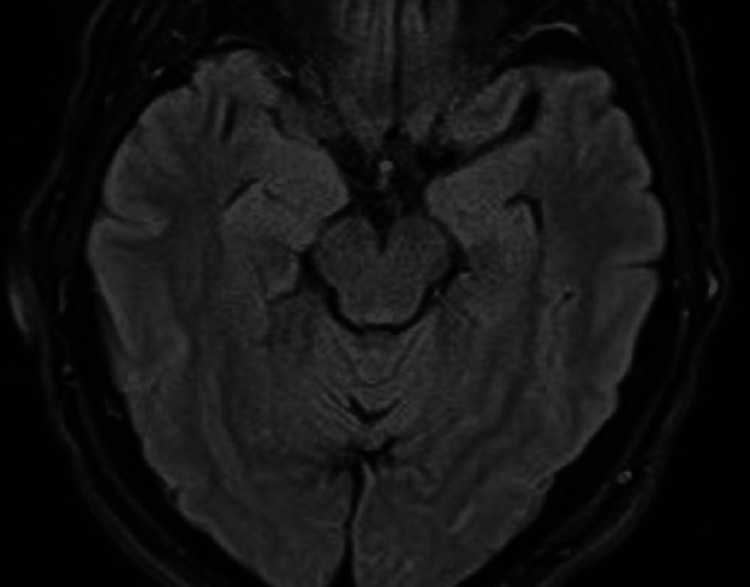
MRI fluid-attenuated inversion recovery (FLAIR) sequence eight months after the episode

**Figure 6:**
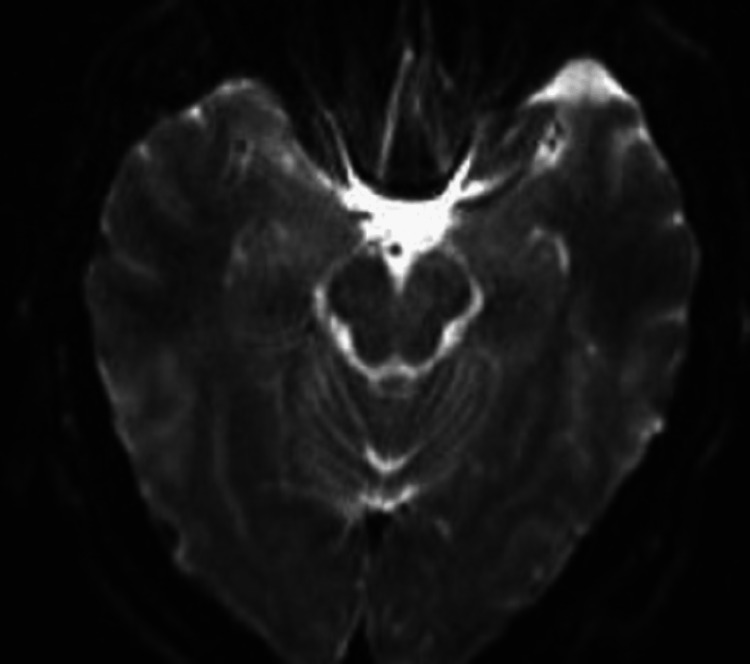
MRI T2-weighted sequence eight months after the episode

The patient followed up three months, four months, and nine months after his hospital admission. In each case, his cognitive (basic orientation, reasoning, and language) and neurological examinations were normal. A follow-up MRI brain scan was performed eight months after his hospital admission and showed resolution of his medial right temporal punctate focus of restricted diffusion with no new infarcts or other intracranial abnormalities present (Figures 4-6). These findings indicated that the patient’s original diagnosis was likely not an ischemic stroke but rather TGA or complicated migraine.

## Discussion

Fisher and Adams described TGA for the first time in 1964, discussing 17 cases with an abrupt onset of anterograde amnesia along with confusion that cleared up in a matter of hours [6]. The diagnostic criteria for this clinical condition were determined by Hodges and Warlow in 1990 and include the following: anterograde amnesia must be observed during the incident, someone must witness the incident, cognitive affection is restricted to amnesia, there is no loss of awareness or personal identity, there are no focal neurological deficits or epileptic symptoms, and the incident must end within 24 hours [7].

Women are affected by TGA more often than men, and the highest incidence occurs during the seventh decade of life when risk factors and accompanying diseases are more common with a peak found at age 62 [8-11].

Most of the cases report difficulties recalling recent memories, with few or no difficulties recalling distant events. Other cognitive skills may be marginally disrupted as well. Several potential triggers have been identified, including physical and highly emotional exertion, exposure to extreme temperatures or high altitude, and slight head trauma. High blood pressure, ischemic heart disease, and migraine have all been identified as potential risk factors. Sexual intercourse has also been specifically identified as a probable trigger. According to a study authored by Fisher, precipitating factors have been identified in 26 out of 85 cases of TGA, with sexual intercourse linked to seven of them. There were also two cases described by Fisher and Adams who experienced TGA at the peak of sexual intercourse [12].

Historically, magnetic resonance diffusion-weighted imaging (DWI) has been utilized to detect abnormalities associated with TGA to help in diagnosis. Evidence of activity in the hippocampal/entorhinal cortex, which is involved in the development of early memories and their transmission to distant memories to be preserved, in the brain has been reported. Lesions are most noticeable 24 to 48 hours after the TGA episode. Our patient had his MRI performed 13 hours after the event, which demonstrated punctate focus of restricted diffusion in the medial right temporal lobe in the region of the hippocampus [13].

Incorporating this within the diagnostic criteria would add a step to the diagnosis and knowledge of TGA pathogenesis. Clinicians can seek to control the events and stop them from happening in the first place. According to a recent study done by Szabo et al., magnetic resonance diffusion-weighted imaging (DWI) has been utilized to identify abnormalities in 390 patients within one to three days of coming to the hospital. A total of 70.6% of TGA patients were found to have hippocampal DWI lesions. They performed an MRI that helped in confirming their clinical certainty. As a result, in these circumstances, this diagnostic criterion is beneficial and is a good addition to the body of knowledge of the condition. Their study has shown that these lesions can be used to diagnose TGA and play a great role in the diagnostic criteria. There is minimal acceptance of standard treatment for TGA since there is no broadly agreed pathogenesis, which may be, in part, due to the short duration of TGA episodes. The treatment is primarily supportive, with IV thiamine sometimes considered. The patient is usually kept in the hospital until the memory loss subsides [13].

Transient global amnesia is an uncommon, benign disease with a favorable prognosis. Recurrence rates were found to be 2.9-23.8% in a population-based study in Olmsted County, MN. Patients with TGA were also compared to controls in a 12-year cohort study, which found no statistically significant changes in transient ischemic attack (TIA), cerebrovascular accident (CVA), seizures, or cognitive abnormalities between the two groups. Ischemia in bilateral hippocampi has been hypothesized as a probable cause of the associated loss of declarative memory. Migraines and epilepsy have been suggested as possible correlates of TGA. Several relationships with other probable triggers have been reported, but none of them has been confirmed widely [14].

## Conclusions

Transient global amnesia is an uncommon, benign disease with a favorable prognosis, which can occur among people who are typically healthy and do not have any comorbidities, risk factors, or triggering factors. Physicians should continue focusing on ruling out other acute conditions like TIA, stroke, seizure, trauma, and infection. The presence of sexual intercourse as a probable triggering component may guide clinicians to an accurate diagnosis in patients with similar histories. More studies are required to investigate the various possible pathways that lead to TGA.

## References

[1] Lin KH, Chen YT, Fuh JL, Li SY, Chen TJ, Tang CH, Wang SJ (2014). Migraine is associated with a higher risk of transient global amnesia: a nationwide cohort study. Eur J Neurol.

[2] Rowan AJ, Protass LM (1979). Transient global amnesia: clinical and electroencephalographic findings in 10 cases. Neurology.

[3] Jaffe R, Bender MB (1966). E.E.G. studies in the syndrome of isolated episodes of confusion with amnesia "transient global amnesia". J Neurol Neurosurg Psychiatry.

[4] Jang JW, Park SY, Hong JH, Park YH, Kim JE, Kim S (2014). Different risk factor profiles between transient global amnesia and transient ischemic attack: a large case-control study. Eur Neurol.

[5] Agosti C, Akkawi NM, Borroni B, Padovani A (2006). Recurrency in transient global amnesia: a retrospective study. Eur J Neurol.

[6] Fisher CM, Adams RD (1964). Transient global amnesia. Acta Neurol Scand Suppl.

[7] Hodges JR, Warlow CP (1990). Syndromes of transient amnesia: towards a classification. A study of 153 cases. J Neurol Neurosurg Psychiatry.

[8] Arena JE, Brown RD, Mandrekar J, Rabinstein AA (2017). Long-term outcome in patients with transient global amnesia: a population-based study. Mayo Clin Proc.

[9] Zhu J, Lu D, Sveinsson O (2015). Is a cancer diagnosis associated with subsequent risk of transient global amnesia?. PLoS One.

[10] Pantoni L, Bertini E, Lamassa M, Pracucci G, Inzitari D (2005). Clinical features, risk factors, and prognosis in transient global amnesia: a follow-up study. Eur J Neurol.

[11] Erkelens CD, Snoek JW (2010). What doctors should not forget about transient global amnesia. Eur J Gen Pract.

[12] Bucuk M, Muzur A, Willheim K, Jurjević A, Tomić Z, Tuskan-Mohar L (2004). Make love to forget: two cases of transient global amnesia triggered by sexual intercourse. Coll Antropol.

[13] Ramjohn NS, Kallan A, Qureshi MA (2022). A case of transient global amnesia: a rare diagnosis. Cureus.

[14] Yi M, Sherzai AZ, Ani C, Shavlik D, Ghamsary M, Lazar E, Sherzai D (2019). Strong association between migraine and transient global amnesia: a National Inpatient Sample analysis. J Neuropsychiatry Clin Neurosci.

